# Observations on Glucose Excursions With the Use of a Simple Protocol for Insulin, Following Antenatal Betamethasone Administration

**DOI:** 10.3389/fendo.2020.592522

**Published:** 2021-01-13

**Authors:** Chané Paulsen, David R. Hall, Deidré Mason, Marí van de Vyver, Ankia Coetzee, Magda Conradie

**Affiliations:** ^1^Department of Obstetrics & Gynecology, Stellenbosch University and Tygerberg Hospital, Cape Town, South Africa; ^2^Department of Medicine, Division of Endocrinology, Stellenbosch University and Tygerberg Hospital, Cape Town, South Africa

**Keywords:** diabetes, pregnancy, betamethasone, insulin, hyperglycemia, hypoglycemia, protocol, LMIC

## Abstract

**Aims:**

Pregnant women with diabetes often require preterm delivery. Antenatal betamethasone reduces perinatal morbidity and mortality, but induces hyperglycemia. The primary objective was to observe glucose excursions and determine the preliminary safety of a protocol for subcutaneous insulin following betamethasone administration in an antenatal ward.

**Material and Methods:**

This retrospective study included all women with diabetes who received betamethasone due to anticipated preterm delivery. Glucose excursions were evaluated in the fasting state and 2-h postprandial. Blood glucose values ≥14mmol/L or ≤3.5mmol/L were regarded as unacceptable hyper- and hypoglycemia respectively. Events over the first 96 h were documented.

**Results:**

This study spanned 52 months and included fifty-nine women. Eleven episodes of defined hypoglycemia occurred in six women, all receiving insulin therapy, but none after a corrective dose of insulin. No serious hypoglycemic incident was reported. Seventeen women experienced hyperglycemic incidents almost entirely (47/56) within 48 h of betamethasone administration, most often postprandially (34/56) and in 85% of episodes, preceded by pre-prandial values >9 mmol/L (29/34). 14 (82.4%) of these women were receiving background insulin therapy. No case with gestational diabetes encountered defined hyperglycemia.

**Conclusions:**

This small study demonstrated preliminary safety of the protocol. Enhanced surveillance is necessary for 72 h after initiation of betamethasone.

## Introduction

Obesity is a major health challenge. Globally, the percentage of adult women who are overweight increased from 29.8% in 1980 to 38% in 2013 (1). In South Africa, more than a third of women are considered obese, and trends of obesity in this population have mirrored the rest of the world (1, 2). Furthermore, women are often delaying pregnancy. Consequently, the pregnant population is now heavier and older than before (3).

Maternal obesity increases adverse pregnancy outcomes such as hypertensive disorders, gestational diabetes, surgical complications, as well as fetal and neonatal complications (4). The prevalence of diabetes in pregnancy is about 16.9% globally (5), being the most common medical disorder of pregnancy (6).

Preterm birth is a global problem (7), with spontaneous or iatrogenic, preterm delivery occurring in up to 20% of pregnancies with diabetes (6). Complications of diabetes in pregnancy associated with early delivery include hypertension, infections, intra-uterine growth restriction, and polyhydramnios. Preterm birth has significant health implications for the newborn such as neonatal death, respiratory distress syndrome, intraventricular hemorrhage, and necrotizing enterocolitis. These infants may be more prone to pulmonary immaturity than infants of non-diabetic pregnancies through an independent disease-related effect (8).

Antenatal corticosteroid administration before preterm delivery dramatically improves perinatal outcomes, being supported by robust clinical trials (7, 9). When preterm delivery threatens a pregnancy complicated by diabetes, it is reasonable to assume that these babies stand to benefit as much, or more, from corticosteroid therapy (10). However, corticosteroids induce hyperglycemia (11). Pregnancy is characterized by relative glucose intolerance and insulin resistance; thus, addition of high dose corticosteroids may cause significant disruption in glucose control in women with diabetes. This hyperglycemia increases the risk of ketoacidosis and may cause fetal acidosis and compromise. Research shows that significant amounts of supplementary insulin are required to control blood glucose levels in the initial period following high dose corticosteroid therapy (10). This raises the question of how supplementary insulin should be administered, whether by continuous intravenous or subcutaneous infusions, or using an adapted basal bolus regimen (12). Ideally, patients should be admitted into a high-dependency setting, with careful maternal and fetal monitoring. Such units are, however, a scarce resource in low- to middle-income countries.

Clinical trials to assess the safety of antenatal corticosteroids have traditionally excluded subgroups of women with confounding conditions, such as diabetes. There is thus a lack of consistent, evidence-based guidance, creating barriers to effective management. A review published in 2016 identified no eligible studies on preterm birth outcomes following antenatal corticosteroid therapy in pregnancies complicated by diabetes (8).

This study aimed to assess the preliminary safety of a local protocol for intermittent subcutaneous insulin following antenatal betamethasone administration to pregnant women with diabetes in a standard ward at Tygerberg Hospital, South Africa.

## Materials and Methods

**Figure 1:**
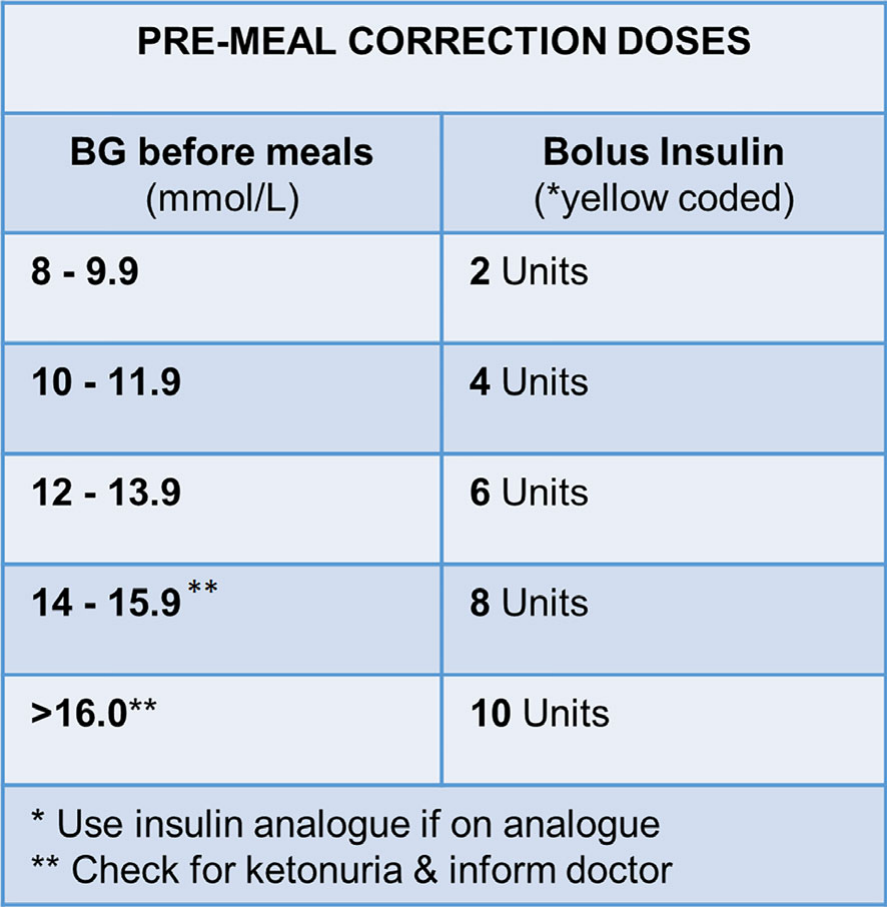
Local protocol for pre-prandial bolus insulin correction doses in management of hyperglycemia in pregnant women with diabetes during betamethasone administration. *yellow coded insulin, short-acting human insulin; BG, blood glucose.

This study was a retrospective audit, in the Obstetric Special Care (OSC) ward at Tygerberg Hospital, a secondary/tertiary referral center. All women with diabetes and at significant risk of preterm delivery were admitted to this unit. The study population included all such pregnancies that received a course of antenatal betamethasone (12 mg IM repeated once after 24 h) and were managed according to the local protocol for pre-prandial corrected subcutaneous insulin (**Figure 1**). No exclusion criteria were identified.

Diabetes was classified as pregestational Types 1 and 2, gestational diabetes and undiagnosed pregestational diabetes detected for the first time in pregnancy. Gestational diabetes was diagnosed using a fasting capillary glucose (CG) level ≥5.6 mmol/L or a 2-h postprandial level ≥7.8 mmol/L. As formal 75 g OGTTs are not performed in our setting, the 2-h value was taken after the patient had consumed her own breakfast. In cases with no history of diabetes, when the fasting CG was ≥7 mmol/L, or the 2-h postprandial level ≥11.1 mmol/L, or the HbA1c ≥6.5% (≥48 mol/mmol), the patient was classified as undiagnosed pregestational diabetes. For further interpretation patients were divided into two main diabetes sub-classes, namely those with Type 1 diabetes and “others”, which included known or suspected Type 2 diabetes and true gestational diabetes.

Treatment before admission included lifestyle modification, metformin, and insulin as appropriate (13). The local protocol for pre-prandial corrected rapid-acting, subcutaneous insulin was applied whatever form of treatment the patient was receiving at the time of inpatient betamethasone administration. Initiation of betamethasone therapy was not confined to any specific time of day. Fasting, pre-prandial, 2-h postprandial, and 02h00 capillary samples were recorded for 96 h thereafter. No venous samples were drawn. Point of care was utilized for the finger prick capillary glucose, and performed on the Accucheck Active (Roche Diagnostics, Mannheim, Germany) glucometer. These hand-held devices determine blood glucose concentration by means of glucose test strips and reflectance photometry and have a measuring range of 0.6–33.3mmol/L. The device is whole blood calibrated; blood glucose values displayed therefore correspond to plasma. The analytical performance has previously been evaluated against rigorous laboratory criteria and did not exceed total allowable error in the fasting or postprandial state (14). The glucose results are reported in mmol/L. Care was also exercised to limit any contamination of the sample. The finger used for the measurement was cleaned with water and thoroughly dried.

Determination of HbA1c was based on the turbidimetric inhibition immunoassay on the Siemens ADVIA 1800 platform. The assay has a measuring range of 0.23%–17.8%, with a reported Coefficient of Variation (CV) of 1.2% at HbA1c level of 5.08% and 2.0% at a level of 10.1% respectively (15 Siemens package insert). HbA1c measurements are reported as a percentage according to the “National Glycohemoglobin Standardization Program” (NGSP) and in mmol/mol units as proposed by the International Federation of Clinical Chemistry and Laboratory Medicine (IFCC)(mmol/mol) units. The HbA1c method is traceable to the NGSP reference method.

The primary aim was to ascertain preliminary safety when antenatal betamethasone was administered to pregnant women with diabetes outside of a dedicated high-care environment. Safety was evaluated based on the documentation of hypo- or hyperglycemia in the fasting state (02h00 and early morning before breakfast) and 2-h postprandial for breakfast, lunch and supper. A CG value ≤3.5 mmol/L indicated hypoglycemia with safety concerns. A CG value ≥14 mmol/L at any of the mentioned time points was regarded as unacceptable hyperglycemia with significant risk for the development of keto-acidosis. Hypoglycemia was further defined using the classification of the American Diabetes Association in their 2018 consensus paper regarding glycemic targets (15). A serious hypoglycemic episode is not linked to any specific glucose threshold, but implies hypoglycemia associated with severe cognitive impairment requiring external assistance for recovery. A blood glucose value below 3 mmol/L is regarded as clinically significant hypoglycemia. Patients manifesting with hyper- or hypoglycemia and the total numbers of hyper- and hypoglycemic events over the first 96 h were documented. Safety was evaluated for the total cohort and the different diabetes types (Type 1 diabetes mellitus, Type 2 diabetes mellitus and gestational diabetes).

Secondary outcomes included the impact of diabetes type (Type 1 DM versus others) on safety, the appropriateness of the surveillance period, the efficacy of the protocol and overall compliance to the protocol. The appropriateness of the surveillance period of 96 h following the first dose of betamethasone was evaluated for the total cohort and the different diabetes subtypes, based on the number of corrective doses. Finally, adherence and compliance with the proposed protocol was assessed based on the number of correction doses given or omitted and if given, how many were accurate in accordance with the protocol.

Data were collected by the principal investigator from electronic and hard-copy records and transferred onto an anonymized data sheet with case numbers only. Three attempts were made before classifying records as lost. Statistical analysis was conducted using the Statistica (version 13, StatSoft) program. The Kolmogorov-Smirnov (K-S) normality test with Lilliefors correction was used (p<0.05) and normal distribution confirmed with the Shapiro-Wilk test. Results are reported as either n (%), means (SD) (normally distributed) and/or median (range) (non-parametric data). In cases where data were not normally distributed, non-parametric Kruskal-Wallis ANOVA with Dunns multiple comparison test was used to determine differences between groups and time. A probability value (p-value) of <0.05 was regarded was significant.

The study was approved with the Human Research and Ethics Committee of Stellenbosch University (S16/10/195).

## Results

The study spanned 52 months (01/11/2013 to 28/02/2018). 62 women with diabetes at significant risk of preterm delivery and/or pulmonary immaturity received an antenatal course of betamethasone in the OSC ward. Two cases were excluded due to irretrievable data and one for protocol violation, leaving 59 for analysis. The descriptive data are given in **Table 1**.

**Table 1:** Baseline characteristics of patients (n = 59).

Description	Mean (SD), median^#^ or n (%)*	Range
*General*		
Age (years)	32.8 (5.8)	20–44
BMI (kg/m^2^)	32.4 (6.9)	20–50
Gravidity^#^	3	1–8
Parity^#^	2	0–6
Gestation - betamethasone (weeks)^#^	30	25–38
*Diabetes class n (%)*		
Type 1	15 (25.4%)	
Other types	44 (74.6%)	
Type 2	28 (47.5%)	
Gestational	7 (11.9%)	
Undiagnosed pregestational	9 (15.3%)	
*Diabetes treatment n (%)*		
Lifestyle only		
Total cohort	2 of 59 (3.4%)	
Type 1	0 of 15 (0.0%)	
Other types	2 of 44 (4.5%)	
Lifestyle + metformin		
Total cohort	17 of 59 (28.8%)	
Type 1	0 of 15 (0.0%)	
Other types	17 of 44 (38.6%)	
Insulin		
Total cohort	40 of 59 (67.8%)	
Type 1	15 of 15 (100.0%)	
Other types	25 of 44 (56.8%)	
*HbA1c before admission*		
NGSP (%)	7.6 (1.5)	4.7–11.2
IFCC mol/mmol	59.6 (11.1)	27.9–98.9
*Comorbid metabolic abnormalities*		
Obesity (BMI ≥30kg/m^2^)	42 of 59 (71.2%)	
Type 1	4 of 15 (26.7%)	
Other types	38 of 44 (86.4%)	
Hypertensive disorders	39 of 59 (66.1%)	
Chronic hypertension	11 of 39 (28.2%)	
Pre-eclampsia	17 of 39 (43.6%)	
Superimposed pre-eclampsia	11 of 39 (28.2%)	
*Other comorbid conditions*		
PPROM or PTL	18 of 59 (30.5%)	
Placental dysfunction	10 of 59 (16.9%)	
Multiple pregnancy	5 of 59 (8.5%)	

HbA1C reported according to the National Glycohemoglobin Standardization Programme (NGSP) as a % and as mol/mmol according to the International Federation of Clinical Chemistry (IFCC). PPROM, preterm pre-labor rupture of membranes; PTL, preterm labor; placental dysfunction, umbilical artery Doppler velocimetry ≥95th centile; Diabetes “Other types” include Type 2 and gestational diabetes, as well as those with undiagnosed pregestational diabetes mellitus.

^#^median; *percentage.

No serious hypoglycemic episode was encountered. Eleven hypoglycemia values (CG ≤3.5 mmol/L) occurred in six patients, with episodes for a single case ranging from 1–3. Four episodes were documented in the fasting state before breakfast, whereas no hypoglycemic values were noted at the 02h00 time point and only three hypoglycemic episodes occurred postprandially (3.4–3.5 mmol/L). None of the latter three episodes occurred after a corrective dose of insulin. Clinically significant hypoglycemia (CG <3 mmol/L) occurred pre-prandially at breakfast and lunch on day three in a single patient with Type 1 diabetes.

Unacceptable hyperglycemic episodes (≥14 mmol/L) occurred in 17 (28.8%) women, with 56 episodes (14 fasting or pre-prandial, 34 postprandial and 8 at 02h00), the range of episodes for a single case being 1–13. The majority of hyperglycemic episodes thus occurred postprandially (60.7%) and were mostly preceded (85.3%) by pre-prandial CG measurements >9 mmol/L. 47 of the hyperglycemic episodes (83.9%) happened during the first 48 h after initiation of betamethasone. Nine hyperglycemic episodes occurred from 49–72 h and none from 73–96 h.

Hypoglycemia occurred in four patients with Type 1 and in two with Type 2 diabetes. The patients with Type 2 diabetes were both on insulin therapy preceding the study. Blood glucose values below 3 mmol/L were confined to a single patient with Type 1 diabetes and were documented pre-prandially at breakfast and lunch (values 2.4 and 2.9 mmol/L) on day 3. Hyperglycemia was documented in six women with Type 1 (6/15; 40%) and in 11 women with other diabetes mellitus subtypes (11/44; 25%). The vast majority of hyperglycemic episodes occurred in insulin-treated patients (14/17; 82.4%). None of the women with true Gestational Diabetes encountered a hyperglycemic episode following betamethasone therapy.

**Figure 2:**
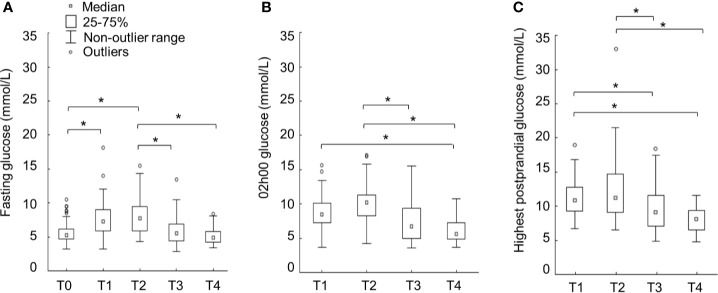
Median capillary glucose values (**A**=fasting; **B**=02h00; **C**=highest postprandial) in women following betamethasone initiation during the surveillance period. T0=before betamethasone, T1 ≤ 24 h, T2 = 25–48 h, T3 = 49–72 h, and T4 = 73–96 h after first dose of betamethasone. Data illustrated as box- and whisker plots (outlier range 95%–5%). *Significance determined with Kruskal-Wallis multiple comparisons test, significance p < 0.05.

The median fasting, 02h00 and highest postprandial values for all cases are shown in **Figure 2**. Postprandial values following breakfast, lunch and supper are tabulated in **Table 2** for the observation period (T1–T4 = 4 x 24-h periods) for all cases (n=59), patients with Type 1 (n=15) and the other diabetes subtypes (n=44) respectively. For the observation period (T1-T4), peak blood glucose values were noted during T1 and T2 (within 48 h after the first and within 24 h after the second betamethasone injection) (**Figure 2**). Blood glucose values returned to baseline on T3 and T4 for the fasting, 02h00 and all postprandial time points (**Table 2**). When subdivided into Type I diabetes versus other diabetes types, there were no significant differences in the fasting, 02h00 and highest postprandial glucose values (not shown).

**Table 2:** Postprandial capillary glucose values over the observation period of 96 h.

Timing and DM subtype	Blood glucose mmol/L: median (range)			
	T1	T2	T3	T4
*Breakfast*				
Total cohort	9.0 (5.3–15.5)	9.7 (4.6–33.3)	6.9 (4.0–17.5)	7.8 (4.1–10.7)
Type 1	11.0 (7.1–14.2)*	11.2 (5.3–15.1)	7.6 (4.1–13.6)	8.9 (4.8–10.7)*
Other types	8.9 (5.3–15.5)*	8.9 (4.6–33.3)	6.3 (4–17.5)	6.5 (4.1–10.5)*
*Lunch*				
Total cohort	8.5 (4.4–15.0)	8.3 (4.4–23.5)	7.3 (3.7–18.4)	6.3 (4.2–10.5)
Type 1	10.2 (4.4–14.0)	9.3 (4.7–15.5)	7.7 (4.4–18.4)	6.4 (5.1–9.8)
Other types	7.8 (4.7–15.0)	7.9 (4.4–23.5)	7.2 (3.7–12.5)	6.2 (4.2–10.5)
*Supper*				
Total cohort	9.8 (3.5–18.9)	10.2 (6.2–21.5)	7.9 (3.4–17.2)	6.7 (3.7–11.6)
Type 1	10.4 (3.5–14.3)	11.8 (7.0–20.0)	6.3 (3.4–13.8)	8.7 (4.1–11.0)*
Other types	9.5 (4.7–18.9)	9.4 (6.2–21.5)	8 (3.5–17.2)	6.4 (3.7–11.6)*

T1 ≤ 24 h, T2 = 25–48 h, T3 = 49–72 h, and T4 = 73–96 h after first dose of betamethasone. Diabetes “Other types” include Type 2 and gestational diabetes, as well as those with undiagnosed pregestational diabetes mellitus. Statistical analysis: Multiple comparisons Kruskal-Wallis ANOVA. *p<0.05 between Type 1 and the other diabetes subtypes at the same time point.

**Figure 3:**
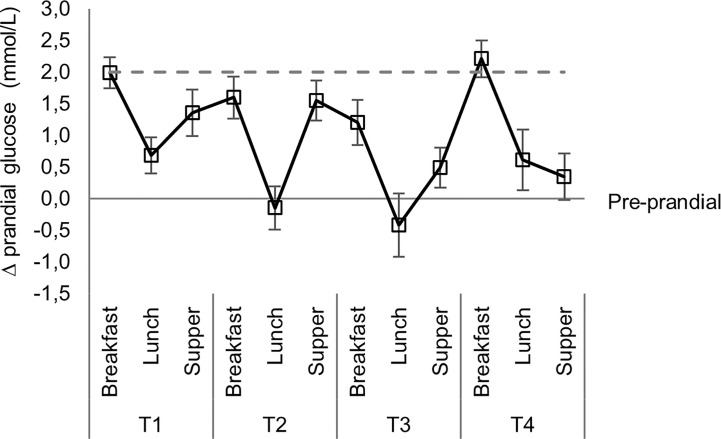
Mean prandial glucose excursions over mealtimes.

Prandial glucose excursions over breakfast, lunch and supper are shown in **Figure 3**. Prandial excursions in excess of 2 mmol/L were limited to breakfast time and only noted during T1 and T4; with the peak average excursion being 2.2 ± 1.7 mmol/L noted during T4.

Overall compliance was 83% (497/598 time-points), ranging between 67% and 95% at different meal-times and during the 24-h observation periods T1–T4 (**Table 3**). Most pre-prandial insulin correction doses were required during T2 (92 doses) followed by T1 (75 doses). The percentage of doses incorrectly omitted varied, with 56% during T1, 36% during T2 and T3 and 48% during T4. The accuracy of the given doses was 92% (120/130 doses given). The majority of missed doses (59%) occurred at the lowest step (8.0–9.9 mmol/L) of CG values prompting correction. Only two episodes of incorrect pre-prandial inulin dosing occurred at values below protocol criteria and in both instances only 2U were given.

**Table 3:** Protocol compliance: Prandial insulin corrections at different time points over 96 h.

	T1	T2	T3	T4
Breakfast				
doses given (n)	8	16	4	2
doses correctly omitted (n)	37	33	46	38
doses incorrectly omitted (n)*	13 (8)	8 (4)	3 (3)	2 (2)
accuracy of given corrections (%)	100%	94%	100%	50%
unknown datasets (n)	1	2	6	17
compliance to protocol (%)	78% (45/58)	86% (49/59)	95% (50/53)	95% (40/42)
Lunch				
doses given (n)	14	18	12	5
doses correctly omitted (n)	25	24	33	30
doses incorrectly omitted (n)*	13 (7)	15 (7)	6 (3)	3 (3)
accuracy of given corrections (%)	92%	100%	92%	60%
unknown datasets	7	2	8	21
compliance to protocol (%)	75% (39/52)	74% (42/57)	88% (45/51)	92% (35/38)
Supper				
doses given (n)	11	25	13	4
doses correctly omitted (n)	21	19	30	29
doses incorrectly omitted (n)*	16 (8)	10 (6)	7 (7)	5 (2)
accuracy of given corrections (%)	100%	92%	100%	50%
unknown datasets (n)	11	5	9	21
compliance to protocol (%)	67% (32/49)	81% (44/54)	86% (43/50)	87% (33/38)

T1 ≤ 24 h after first betamethasone, T2 = 25–48 h after first betamethasone, T3 = 49–72 h after first betamethasone, T4 = 73–96 h after first betamethasone. *Number of doses incorrectly omitted in parenthesis refers to omitted corrections with pre-prandial glucose values between 8–9.9mmol/L.

The median (range) gestation at delivery was 34 (27–38) weeks, while the mean birthweight was 1,472 (SD ± 490) grams. Only two babies (2%) had an Apgar score of less than seven, at 5 min. There were no intra-uterine deaths.

## Discussion

Pregnant women with diabetes regularly face early preterm delivery. Antenatal betamethasone improves perinatal outcome, but elevates blood glucose necessitating careful management (16). A systematic review in 2016 lamented the lack of evidence regarding safety of corticosteroids for women with diabetes (8).

No serious hypoglycemia episode was observed in this study. Those on insulin were more likely to develop hypoglycemia but no case with true gestational diabetes developed hypoglycemia. Defined hyperglycemia was encountered more readily, but never resulted in metabolic decompensation. Hyperglycemia occurred predominantly within the first 48 h after initiation of betamethasone. Special care is therefore advised during this period, especially in women already receiving insulin. As hyperglycemia may occur repeatedly, higher risk cases should be monitored more closely. The pre-prandial protocol was not devised to correct prior poor glycemic control in individuals, but rather to safely limit glucose elevations caused by betamethasone.

Short-acting, subcutaneous insulin was used for the sake of simplicity. This contrasts with supplementary intravenous insulin infusions, also in wards by Kaushal et al., who required large amounts to achieve even moderate glycemic control (10). Similarly, a Diabetes UK Position Statement advocates that hyperglycemia following steroid administration be managed by variable rate intravenous- or continuous subcutaneous insulin infusion (12). Most hyperglycemic episodes occurred postprandially and was almost invariably preceded by elevated pre-prandial values. Median prandial excursions were modest and within the allowed 2 mmol/L increase for all mealtimes. These observations would support a conservative increase in basal insulin dosages during the T1 and T2 observation periods in women already on insulin, rather than intensifying the pre-prandial insulin correction protocol with potential excessive prandial lowering in blood glucose values. No hypoglycemia was documented at 02h00 and the median glucose values for the total cohort and diabetes subtypes at this time point, argue that a conservative increase in basal insulin during the first 48 h, usually administered at bedtime, will be safe. This should especially be considered in patients presenting with hyperglycemia prior to betamethasone administration and those who manifest with pre- and postprandial hyperglycemia, early in the observation period. In describing their algorithm for insulin-dependent women receiving glucocorticoids for fetal lung maturation, Mathiesen et al, noted the individual doses of prandial and basal insulin before glucocorticoid injection. Then, for 7 days, the doses of both insulin types were increased (up to 40% on days two and three) and thereafter gradually decreased back to pre-glucocorticoid levels (17). As in the index study, no cases of ketoacidosis or severe hypoglycemia were described.

The duration of enhanced surveillance and dose adjustment is an important issue for institutions with a high demand for beds and limited resources. Mathiesen et al., described one week, while Kalra et al., proposed at least five days (18). In the index study the most important period was the first 48 h following the betamethasone initiation. The limited number of insulin corrections required during T4 and the absence of hyperglycemic episodes argues for the shortening of the observation period to 72 h, especially in resource-constrained settings, without adversely impacting safety. This study has limitations. It was a fairly small, retrospective, pilot-type study and therefore had no power calculation. On the other hand, the paucity of evidence regarding safety of corticosteroids for pregnant women must be addressed. Further strengths are that that the study was performed in a standard (not high-care) ward, using a simple, reproducible protocol in a middle-income country. Although the protocol was intentionally simple, the importance of strict adherence to pre-prandial insulin corrections following betamethasone must be emphasized to caregivers, especially in a standard ward during the time periods mentioned.

## Data Availability Statement

The raw data supporting the conclusions of this article will be made available by the authors, without undue reservation.

## Ethics Statement

The studies involving human participants were reviewed and approved by the Human Research and Ethics Committee of Stellenbosch University.

## Author Contributions

DH and MC conceptualized the study. CP captured the data with assistance from DM. All co-authors contributed to the manuscript, while MV performed the statistical analysis assisted by MC and DH. All authors contributed to the article and approved the submitted version.

## Conflict of Interest

The authors declare that the research was conducted in the absence of any commercial or financial relationships that could be construed as a potential conflict of interest.
